# Adaptive Working Memory Training Can Improve Executive Functioning and Visuo-Spatial Skills in Children With Pre-term Spastic Diplegia

**DOI:** 10.3389/fneur.2020.601148

**Published:** 2021-01-20

**Authors:** Maria Chiara Di Lieto, Chiara Pecini, Paola Brovedani, Giuseppina Sgandurra, Marta Dell'Omo, Anna Maria Chilosi, Andrea Guzzetta, Silvia Perazza, Elisa Sicola, Giovanni Cioni

**Affiliations:** ^1^Department of Developmental Neuroscience, Istituto di Ricovero e Cura a Carattere Scientifico (IRCCS) Fondazione Stella Maris, Pisa, Italy; ^2^Department of Education, Language, Interculture and Psychology, University of Florence, Florence, Italy; ^3^Department of Clinical and Experimental Medicine, University of Pisa, Pisa, Italy

**Keywords:** pre-term spastic diplegia, executive function, visuo-spatial function, neuropsychological training, cogmed working memory training

## Abstract

Pre-term spastic diplegia (pSD) due to periventricular leukomalacia is a form of cerebral palsy in which weaknesses in executive functions are reported beyond the core visuo-spatial deficits. The study aimed at improving executive functioning and visuo-spatial skills with an evidence-based training focused on working memory in children with pSD. The intervention study followed a stepped wedge design. 19 children with pSD (11 female and 8 male; age range: 4;1–13;1 years), mild to moderate upper limb impairment and Verbal Intelligence Quotient (VIQ) >80 participated to the study. The children were trained with a home-based adaptive working memory training (CogMed®) over a 5-week period. The primary outcome measure was the CogMed Improvement index; pre- and post-test explorative neuropsychological assessment was conducted with a subset of tests from the NEPSY-II battery. Working memory training in children with pSD significantly improved trained working memory abilities (CogMed indices) as well as non-trained skills, such as visuo-spatial skills, inhibition of automatic responses and phonological processing. The results suggest that standard rehabilitation schedules for children with pSD should be integrated with trainings on executive functions.

## Introduction

Working memory (WM) is the ability to manipulate and update information maintained in memory for brief periods of time (1). It is important in several complex cognitive functions, such as academic learning, planning and organization of daily life activities. School-based activities, indeed, such as math and reading, depend on the ability to hold and integrate several instructions or information in mind (2–8). Working memory belongs to the family of top-down mental processes known as executive functions (EFs) (9, 10). Within a general pattern of shared but distinct EFs (11), the developmental model proposed by Diamond (7) is largely adopted to describe three main EF components: (i) inhibition, the ability to suppress automatic behaviors, memories and thoughts in favor of goal-appropriate responses, (ii) WM, the ability to actively manipulate relevant information in memory and (iii) cognitive flexibility, the ability to switch between two or more tasks, mental sets or response rules. Although it is well-accepted that there are several processes within the EF domain (11), recent reviews of EF interventions highlight that especially WM and inhibition must be continually challenged as they are considered “tools for learning” [(12), page 363], favoring the development of other cognitive skills (6, 8, 12, 13).

Since EF deficits are typically found in children with Attention Deficit and Hyperactivity Disorder [ADHD, for a review see (14)] or traumatic brain injury (15), several studies suggest that EF impairment may be part of different neurodevelopmental disorders such as specific learning disabilities [for a meta-analysis see (16)], specific language impairment [for a review see (17)], intellectual disabilities (18, 19), autism spectrum disorders (20) and cerebral palsy, including spastic diplegia (21, 22).

Spastic diplegia is a form of cerebral palsy (CP) in which both sides of the body are involved, with a predominance to the lower limbs (23). It commonly occurs in preterm born children (pre-term spastic diplegia, pSD) and it is generally due to periventricular leukomalacia, a form of white matter brain injury typically affecting neural pathways lying close to the lateral ventricles, as the corticospinal tract and the optic radiations (24–28). Children with pSD consistently present with impaired non-verbal intelligence and visuo-perceptual and visuo-spatial abilities, while general verbal skills, as reflected by verbal Intelligence Quotient and Indices, are generally spared (26, 28–30). Beyond visuo-spatial deficits and impaired non-verbal intelligence, weak EFs have been also reported in children with pSD, such as the inability to quickly process, maintain, update and inhibit information (21, 31–36). This might be due to the involvement of white matter associative fibers altering brain structural connectivity (24, 26–28), as shown, for example, in children with unilateral periventricular leukomalacia where the altered connectivity to the anterior cingulate cortex is strongly related to EF deficits (37). In a previous study from our group (36), a multilevel organization of the neuropsychological profile in children with pSD was suggested. Beyond the common core visuo-spatial and sensory-motor deficits, when pSD was associated with thinning of the anterior/middle corpus callosum, impairments in attention and EFs seemed to act as additional factors in further affecting visuo-spatial, sensori-motor and social skills.

Despite the complex neuropsychological deficit patterns of pSD, as for other forms of CP, the majority of interventions for CP have been mainly focused on body motor functions and less is known on their effects on cognition, academic achievements and daily life skills [for a review of intervention research see (38)]. Some studies reported, in children with CP, intervention-related improvements in cognitive skills (39, 40) or in psychological well-being and social participation (39, 41–43). Evidence of training effects specifically targeting EFs is only relatively recent and scant, and has been reported for unilateral spastic CP (44, 45) but no studies to date have been performed in children with pSD.

One of the most widely used EF training programs in the scientific literature is the CogMed Working Memory Training (RoboMemo®, CogMed Cognitive Medical Systems AB, Stockholm, Sweden), an evidence-based tele-rehabilitation software, comprising several intensive visuo-spatial and verbal working memory exercises that automatically adapt the level of difficulty to the individual child's performance. In healthy adults, training effects of CogMed have been linked to significant increases of activity in parietal and pre-frontal regions on WM task-related functional Magnetic Resonance Imaging (fMRI) (46–48), supporting training-induced plasticity of the neural systems underlying WM. CogMed's home-based videogame is currently applied to different clinical populations. It has been shown to improve WM skills in children with ADHD (49–52), acquired brain injury (53) as well as in pre-term-born children. In the latter, a population at higher risk for neurodevelopmental delay and CP (54), five non-randomized trials were performed (55–59), all using the CogMed platform. Benefits of the intervention were found in trained WM tasks as well as in untrained memory tasks, in very-low and extremely-low birth weight children, especially in visuo-spatial memory (spatial span and memory for faces tasks) and in verbal short and long term memory tasks (55–57). Beyond the generalized memory improvement, the effect on untrained cognitive processes, such as auditory attention and phonological awareness found by Grunewaldt et al. (56), was not confirmed by another study, most likely due to the methodological differences between the studies (58).

Generalization effects of CogMed training are still debated as some studies have found positive effects (13, 49, 53, 60–64) while others have reported no generalized enhancements of untrained skills (59, 65). The few follow-up studies available report a partially sustained training effect after 3 and 12 months (65, 66).

The present study was aimed at determining the effects of WM training in children with pSD due to periventricular leukomalacia. As working memory represents a fundamental component of EF, it was hypothesized that improving WM may have cascade effects on the deficits classically reported in this clinical population, such as visuo-spatial or sensori-motor impairment. CogMed was chosen as it allows auto-adaptive and intensive exercises at home, reducing the number of hospital visits, which are very frequent for children with pSD. This study replicated the methods and procedures of a previous study on CogMed training on pre-term born children without neuroanatomical lesions (56). The study has been registered with ClinicalTrials.gov, number NCT02342990, on January 20, 2015.

## Materials and Methods

### Participants

Sample size was calculated by expected effect size method (67) by G^*^Power 3 program (68). A total sample size of 19 children was selected, based on the data from 53, showing an effect size on the primary outcome measure *d* = 0.8, alpha = 0.05 and power = 0.95 for a dependent-sample t-test (critical *t* = 1.73) (69).

Nineteen children (11 females, 8 males) with pSD (mean age 7;3 years, SD: 2;4 range 4;1–13;1 years) and a mean gestational age at birth of 31 weeks (range: 28–35 weeks) were selected from a group of 30 children with a CP, recorded as spastic diplegia according to Bax et al. (70), recruited from March 2014 to November 2015 at the Department of Developmental Neuroscience of IRCCS Fondazione Stella Maris. Children were selected according to the following inclusion criteria: (a) neuroradiological diagnosis of periventricular leukomalacia documented at brain MRI performed after age 2 years (by images or on neuroradiological reports); (b) mild to moderate functional upper limb impairment (from level I to III) at the Manual Ability Classification System- MACS (71); (c) absence of drug-resistant epilepsy; (d) absence of a psychiatric disorder diagnosis or sensory deficits that preclude testing; (e) Verbal IQ >80, as assessed in the last year prior to recruitment by WPPSI-III (72), WISC-III (73) or WISC-IV (74). The majority of children (17 out of 19) had significantly higher verbal intelligence than non-verbal. After the enrollment, the children were randomly split into two groups (Cluster A, *n* = 10 and Cluster B, *n* = 9), for sequential rollout of the training. All children were native Italian speakers of European ethnicity and followed care as usual motor rehabilitation. A subset of the children included were part of a previous study (36).

The research project was approved by the Ethical Committee of the Institute (n° 13/2013). Written consent for participation was obtained from all participants' parents who also gave informed consent to publication of results.

### Motor and Visual Assessment

The Gross Motor Classification System (GMCS) (75) was used to determine gross motor skills. Children were classified in five motor levels: walk without restriction (level I); walk without assistive devices but limitation in walking outdoors (level II); walk with assistive mobility devices (level III); self-mobility with limitations (level IV); self-mobility is severely limited even with use of assistive technology (level V). Manual ability was classified according to the MACS (71). Children were classified according to five motor levels: handles objects easily and successfully (level I); handles most objects but with somewhat reduced quality and/or speed of achievement (level II); handles objects with difficulty; needs help to prepare and/or modify activities (level III); handles a limited selection of easily managed objects in adapted situations (level IV); does not handle objects and has severely limited ability to perform even simple actions (level V). Visual functions were derived on the basis of chart report data from the Vision Laboratory of our Department and were assessed for the presence of the following visual deficits: stereopsis impairment, deficits in ocular motility, visual field or visual acuity. Children were classified as follows: normal, absence of deficits; mildly impaired, one or two visual deficits; severely impaired, three or more deficits.

Motor, visual, and cognitive functions of the sample are reported in Table 1.

**Table 1:** Clinical characteristics of the study group of children with pSD.

	**Sex**	**GA**	**Age (y;m)**	**Motor function**		**Visual function deficit**	**Intelligence**	
				**GMFCS level**	**MACS level**		**VIQ**	**PIQ**
**Cluster A**								
S1	M	28	9;0	III	II	Mild	102	85
S2	M	28	6;0	II	II	NA	88	93
S3	F	29	4;1	III	I	NA	106	76
S4	M	31	5;1	III	III	Mild	108	82
S5	F	29	5;1	IV	III	Mild	114	104
S6	F	29	7;0	II	I	Mild	94	95
S7	M	35	6;1	III	I	No	92	61
S8	F	31	5;1	II	II	No	100	82
S9	F	32	8;0	II	III	Mild	82	58
S10	M	30	8;7	II	I	Mild	112	82
***Mean*** ***SD***		***30.1*** ***2.1***	***6***;***7*** ***1***;***7***				***99.8*** ***10.6***	***81.8*** ***14.3***
**Cluster B**								
S11	M	31	8;1	II	II	Mild	104	87
S12	F	32	9;1	III	II	Mild	102	100
S13	F	32	6;1	IV	III	Mild	100	89
S14	M	30	11;0	II	II	Mild	103	59
S15	F	32	6;0	II	II	No	100	80
S16	F	34	13;1	II	II	Mild	98	93
S17	F	32	4;1	II	II	Mild	112	91
S18	M	32	7;8	II	II	Mild	100	62
S19	F	28	9;7	III	III	Mild	99	89
***Mean*** ***SD***		***31.4*** ***1.7***	***8;0*** ***3***;***0***				***102.0*** ***4.2***	***83.3*** ***14.0***

*GA, gestational age; GMFCS, Gross-Motor Function Classification System; MACS, Manual Abilities Classification System; NA, not available; No, normal visual function; VIQ, Verbal IQ at WISC-III, WPPSI-III, or Verbal Comprehension Index at WISC-IV; PIQ, Performance IQ at WPPSI-III or Perceptual Organization Index at WISC-III or Perceptual Reasoning Index at WISC-IV*.

All children with pSD had more impaired lower limbs (GMFCS, classification ranging from Level I to IV) than upper (MACS, inclusion criterion). Visual functions were mildly impaired in the majority of children (14/17).

### Intervention Program

CogMed Working Memory Training (RoboMemo®, CogMed Cognitive Medical Systems AB, Stockholm, Sweden) contains a variety of computerized, game-format tasks that are home-based and auto-adaptive; that is, for each task, the level of difficulty is adjusted automatically to the WM span of the child. This training is available in three on-line versions depending on the child's age. Sixteen children used the school age version (CogMed RM), while three (S3, S8, and S17 in Table 1) used the pre-school version (CogMed JM). Pre-school children who read letters and numbers, at a preliminary qualitative assessment, used the school-age CogMed version. CogMed RM includes 12 visuo-spatial and verbal tasks, eight tasks are provided for each training session for 45 min a day; CogMed JM consists of seven visuo-spatial and verbal tasks for 20 min a day. A training period of 5 weeks, for a total of 25 sessions, was performed by each child at home. A certified coach (MCDL) introduced the CogMed program to the child and his/her family, establishing with them reward systems, goals and treatment planning and followed the training progress weekly calling the families to give advice based on the uploaded results. After the training, two indices were automatically provided by the program: CogMed improvement index, to measure working memory improvement, and CogMed progress indicators, which assesses visuo-spatial and verbal WM span [adapted from the Adaptive Working Memory Assessment; (76)]. For a detailed description of CogMed Working Memory Training see www.cogmed.com/program.

### Study Design

**Figure 1:**
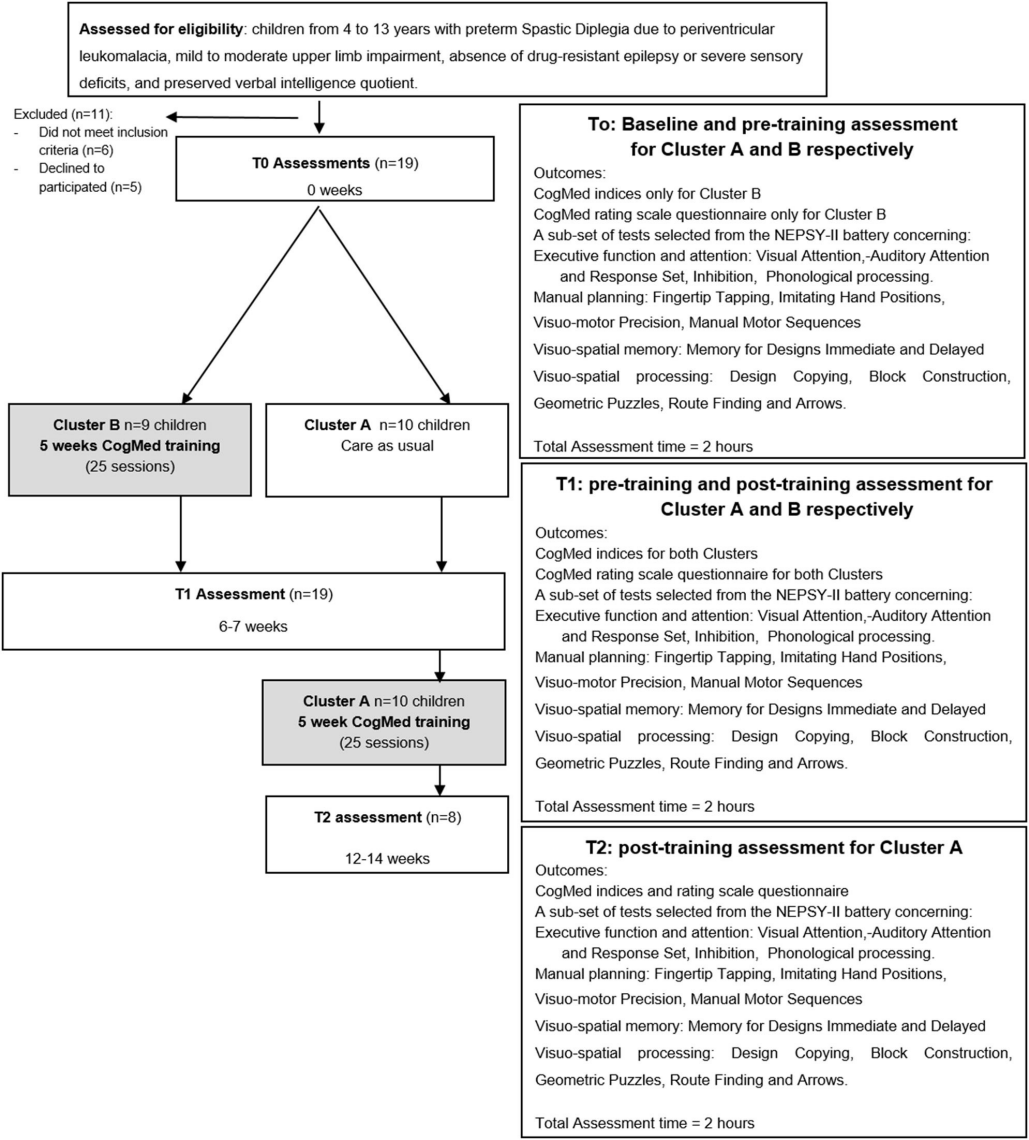
CONSORT flow chart of the study.

As shown in Figure 1, the Stepped Wedge randomized trial design (77) adopted, was the same design previously used by Grunewaldt et al. (56) to study the CogMed effects in a group of premature pre-school children. Thus, the children were randomly split into two groups (Cluster A, *n* = 10 and Cluster B, *n* = 9), for sequential rollout of the training. Both Clusters were assessed with neuropsychological tests from the NEPSY-II at time point T0. Then children in Cluster B immediately started CogMed training, while those in Cluster A did not receive any training in the same period. Six/seven weeks later, all children (Cluster A and B) were retested (time point T1). At T1 Cluster A started CogMed training and 6/7 weeks later was retested at time point T2.

Test-retest effect was calculated comparing performance at T0 and T1 time points in Cluster A. The training effect was evaluated comparing pre- and post- training performance at NEPSY-II in all children (both Cluster A and Cluster B), controlling training differences between Clusters.

### Primary Outcome Measure

The primary outcome measure was the CogMed Improvement index provided by the program, as it is largely used in literature and is representative of the main target of the training, that is, changes in working memory span (53, 56, 78). It is calculated by subtracting the Start Index (the mean of the three best accurate trials on days 2 and 3) from the Max Index (the mean of the three best accurate trials during the training period). The mean CogMed Improvement Index for children aged 7–17 years is 27 (normal range 14–40). For further details about the training intervention and algorithm see CogMed JM and RM; www.cogmed.com and Klingberg et al. (79).

### Explorative Outcome Measures

The CogMed progress indicators assess visuo-spatial and verbal WM span with two tests, Working Memory and Following Instructions, presented by the program at the beginning, the middle and the end of the training. The Working Memory test requires the child to identify a different shape from a set of three and remember its location; in the Following Instruction test the child listens to a set of instructions and then clicks on or drags objects seen on the screen in a specific order. Both tests scores are expressed in span scores.

The CogMed rating scale questionnaire was filled out by the parents as it is aimed at monitoring the child's behavior before and after the training period. The questionnaire collects quantitative data, expressed as raw scores, on inattention, hyperactivity and impulsivity. This questionnaire was filled only by parents of school-aged children, as provided by CogMed.

For pre- and post- neuropsychological evaluation, a sub-set of tests selected from the NEPSY-II battery was chosen (80), with particular attention to those subtests found impaired in a previous study in children with pSD (36). They tapped the following neuropsychological areas: executive function and attention, manual planning, visuo-spatial memory and visuo-spatial processing. Performance was expressed as raw scores, which, if not otherwise specified, were referred to response accuracy, that is the number of correct responses given for each subtest. The following subtests comprised the Executive function and attention assessment:

- Visual Attention: visual search task requiring to cross out one or two targets among a variable number of distractor stimuli;
- Auditory Attention and Response Set: sustained auditory attention task requiring to shift and update new and complex set of rules involving the inhibition of previously learned responses;
- Inhibition: task requiring to inhibit automatic responses in favor of novel responses and to switch between response types. The test is divided into three conditions: naming, inhibition, and switching, but only the inhibition condition was reported. Both accuracy (number of errors) and speed are recorded for each condition;
- Phonological processing: phonemic awareness task requiring to identify pictures corresponding to given word segments and to create new words by omitting or substituting a syllable or a phoneme.

For the Manual planning assessment, the following subtests were administered:

- Fingertip Tapping: tasks requiring to imitate a series of finger movements (single and sequences) with the dominant and non-dominant hand. Speed is recorded;
- Imitating Hand Positions: visuo-motor planning task requiring to imitate finger positions;
- Visuo-motor Precision: visuo-motor integration task requiring to draw a line following paths of different widths and spatial complexity. Both accuracy (number of errors) and speed are measured;
- Manual Motor Sequences: visuo-motor planning task requiring imitation of a series of hand movements.

The Visuo-spatial memory assessment comprised the following subtests:

- Memory for Designs: visuo-spatial memory task requiring to identify form and position of an abstract design on a grid with 4–10 distractors. Content (visual form recognition) and spatial (localization) scores are obtained. Memory for Design Delayed is administered 15–25 min later.

For assessing Visuo-spatial processing, the following subtests were administered:

- Design Copying: visuo-motor integration task requiring to copy geometric figures of increasing complexity;
- Block Construction: constructional praxis task requiring imitation of three-dimensional block constructions of increasing complexity starting from either a three- or a two-dimensional model;
- Geometric Puzzles: mental rotation task requiring to recognize rotated geometric shapes among a series of distractors.
- Route Finding: visual spatial relations and directionality task requiring to find the house, previously shown in a schematic map, in a larger map with other houses and streets.
- Arrows: judgement of line orientation task requiring to find the arrow(s) pointing to the center of a the target in an array of arrows arranged around the target.

### Statistical Analysis

The Statistical Package for Social Sciences, version 17.0 (IBM SPSS Statistics, IBM Corporation, Armonk, NY) was used for statistical analyses.

Normality of distributions was verified by Shapiro-Wilk's test and *t*-tests or non-parametric tests were used according to normal/non-normal distributed data or to continuous/ordinal variables, respectively.

To verify the absence of clinical and performance differences at pre-training assessment and to examine test re-test effect within Cluster A, two-tailed unpaired *t*-tests were conducted on pre-training performance between Cluster A and Cluster B and two-tailed paired *t*-tests within Cluster A.

To test the training effect on primary and explorative outcome measures, Mixed ANOVAs, with Cluster as between-subject factor (A vs. B) and time as repeated factor were performed on the primary outcome measures (CogMed indices) and on the neuropsychological subtest at NEPSY-II. For multiple comparisons, the Bonferroni's correction was applied. Moreover, to determine the effect size, Cohen's d were calculated by G^*^Power 3 program (68).

To verify change within CogMed progress indicators and questionaires, Wilcoxon Signed Rank test were performed comparing performance across the beginning, the middle and the end of the training in the CogMed progress indicators and between pre- and post- training in the CogMed rating scale questionnaire. To describe the relationship between clinical factors and training effects, Parametric or non-parametric bi-variate correlations between clinical characteristics (VIQ, PIQ, chronological age, gestational age, GMFCS, and MACS) and CogMed Improvement index or the degree of improvement for each neuropsychological subtest were performed.

## Results

### Neuropsychological Characteristics of the Two Clusters at Pre-training Assessment

There was no difference in chronological age (*t*(17) = −1.2, ns), gestational age (*t*(17) = −1.4, ns) and gender (χ^2^(1) = 2.7, ns) between the two Clusters.

At T0, no significant differences between Cluster A and Cluster B were found in verbal and non-verbal intelligence (*t*(17) = −0.6, ns; *t*(17) = −0.2, ns respectively) and in GMFCS (χ^2^(3) = 1.6, ns). No differences between the two Clusters were found in Start CogMed index (*t*(15) = −0.3; ns) nor in any other NEPSY-II neuropsychological subtest at pre-training assessment, except for Auditory Attention (*t*(15) = −2.4, *p* < 0.05) and Design Copy (*t*(16) = −2.8, *p* < 0.05) subtests, which, thus, were excluded from further analysis.

### Training Effects on the CogMed Indices

Two children (S7 and S10), included in Cluster A, did not complete the training due to inconsistent family compliance and thus their performance was used only to verify practice test-retest effects. All the other children (*n* = 17) completed the 25-day training period and were tested at all scheduled time points.

As shown in Table 2, the Max Index was significantly higher than the Start Index (*F*_(1, 15)_ = 52.72, *p* < 0.001), without significant Cluster's effect (*F*_(1, 15)_ = 0.26, *p* > 0.05) and Cluster × Time effect (*F*_(1, 15)_ = 0.55, *p* < 0.05), and a large effect size was found (*d* = 1.29). The mean Improvement Index, that did not correlate with the Start and Max indices (*r*(17) = 0.4, ns), was indeed higher (mean 25.2; SD 13.9; range between 8 and 52) than the improvement cut-off value (cut off >14).

**Table 2:** CogMed working Memory indices.

	**Start Index**	**Max Index**	**Improvement Index**
S1	64	83	19^*^
S2	73	125	52^*^
S3	33	41	8
S4	48	63	16^*^
S5	54	76	22^*^
S6	42	78	35^*^
S8	39	54	14^*^
S9	67	80	14^*^
S11	61	77	16^*^
S12	77	101	24^*^
S13	66	79	12
S14	69	95	26^*^
S15	55	89	34^*^
S16	78	128	49^*^
S17	45	67	22^*^
S18	36	52	16^*^
S19	51	101	50^*^
***Mean*** ***(SD)***	***56.3*** ***(14.4)***	***81.7*** ***(23.6)***	***25.2*** ***(13.9)***

* *Significant improvement (≥1 SD from mean)*.

The span scores in the Following Instruction test were significantly higher at the last session with respect to both the beginning (*Z* = −2.8; *p* < 0.005) and the middle (*Z* = −2.3, *p* < 0.05) sessions. The span scores in the Working Memory tests and the behavioral profile at the parent rating scales (filled out for the sub-sample of children who performed the school age CogMed version, *n* = 14) did not significantly change after the training (Wilcoxon signed ranks tests *Z* from −0.14 to −1.9, ns).

### Training Effects on Neuropsychological Measures

Within Cluster A, a test-retest effect was found in Inhibition accuracy (*t*(8) = 3.2, *p* < 0.05), Finger Tapping (*t*(7) = 3.1, *p* < 0.05), Manual Motor Sequences (*t*(9) = −2.6, *p* < 0.05). Thus, in order to avoid test-retest biases, the scores of these subtests were not used to test the training effects.

Mean and SDs for each NEPSY-II subtest, together with the mixed ANOVAs results are presented in Table 3. Within the executive function and attention subtests, significant improvements were found in Inhibition speed, with a moderate effect size, and in Phonological Processing with a small effect size. At the Memory for Design subtest (immediate condition) better performance at the end of the training was found with a moderate effect size, which was non significant after Bonferroni's Correction. Among the visuo-spatial processing subtests, significant improvements were found in Block Construction with a large effect size. No signficant Cluster effect were found, and Cluster × Time effect were found only in Phonological Processing subtests, which already showed almost significant differences between Clusters (*p* = 0.06) at pre-training assessment. A small minority of the children were unable to complete the neuropsychological assessment for clinical reason. The total number of children for each subtest is indicated in Table 3 under the heading “number of children who improved”.

**Table 3:** Comparison between pre- and post- training performance at single subtests.

**Outcome**		**Pre-training Mean (SD)**	**Post-training Mean (SD)**	***F***	**df**	***p***	**Cluster's effect (*p*)**	**Cluster × Time effect (*p*)**	**Cohen's d**	***n*. of children improved**
Executive function and attention	Visual Attention	4.5 (11.7)	7.5 (12.7)	3.29	1, 14	0.091	0.428	1.000	0.2	12/16
	Response Set	27.7 (8.5)	30.4 (6.4)	5.61	1, 6	0.065	0.757	0.065	0.3	4/7
	Inhibition Speed	137.6 (48.7)	122.9 (44.0)	18.08	1, 13	0.001^*^	0.596	0.852	0.3	13/15
	Phonological processing	33.0 (11.1)	34.3 (12.4)	13.65	1, 13	0.003^*^	0.124	0.004^*^	0.1	9/15
Visuo-spatial memory	Immediate	73.3 (37.1)	84.3 (41.0)	7.16	1, 15	0.017	0.317	0.317	0.3	11/17
	Delay	23.3 (12.2)	25.4 (14.3)	2.22	1, 13	0.160	0.103	0.103	0.2	9/15
Sensori-motor skills	Imitation hand position	10.5 (4.7)	11.5 (4.6)	2.55	1, 13	0.134	0.636	0.636	0.2	10/15
	Speed in Visuomotor precision	108.4 (48.6)	116.0 (54.1)	1.82	1, 12	0.202	0.795	0.795	0.2	4/14
	Accuracy in Visuomotor precision	59.4 (52.3)	56.8 (50.7)	0.012	1, 12	0.915	0.215	0.215	0.1	8/14
	Geometric Puzzle	17.8 (7.1)	19.7 (6.1)	9.78	1, 14	0.007	0.209	1.000	0.3	11/16
	Route Finding	3.5 (3.5)	4.7 (3.5)	6.94	1, 11	0.023	0.097	0.162	0.3	9/13
	Block Construction	8.0 (2.5)	9.8 (2.3)	28.29	1, 13	0.001^*^	0.863	0.112	0.7	13/15
	Arrows	14.5 (9.8)	15.5 (7.2)	1.50	1, 12	0.245	0.303	0.886	0.1	9/14

* *Statistical significance after Bonferroni's Correction (p < 0.004) at Mixed ANOVAs for Time effect (pre- vs. post- training assessment), Cluster effect (A vs. B) and Cluster × Time effect (interaction between Cluster and Time variables). n.of children improved: the number of children with improved performance at the end of the training respect to the total number of children who had completed the subtest*.

No significant correlations between CogMed Improvement index and verbal (*r*(17) = −0.3, ns) or non-verbal (*r*(17) = 0.4, ns) intelligence levels were found. Chronological age positively correlated with Start and Max indices (*r*(17) = 0.6, *p* < 0.01; *r*(17) = 0.6, *p* < 0.01, respectively), but not with Improvement index (*r*(17) = 0.40, ns) and no correlations were found between other clinical characteristics and CogMed indices.

Concerning the relationship between clinical characteristics and improvements in the neuropsychological profile, GMFCS level was positively correlated with the immediate Memory for design (rho(17) = 0.63, *p* < 0.005) and negatively with the Arrows (rho(14) = −0.70, *p* < 0.01) subtests. Performance IQ was positively correlated with the improvement in Inhibition Speed (*r*(15) = 0.6, *p* < 0.005) subtest, while verbal IQ was negatively correlated with Response Set (*r*(8) = −0.9, *p* < 0.01) and Imitation hand position (*r*(15) = 0.6, *p* < 0.05) subtests. No correlations were found between gestational age and MACS levels and any neuropsychological subtests.

## Discussion

The main finding of our study is the demonstration that, in children with pSD, a home-based and self-adaptive WM training can improve targeted WM abilities as well as other non-targeted neuropsychological functions, such as visuo-spatial processing, inhibition, and phonological processing. In agreement with previous studies conducted in different clinical populations, we showed, for the first time, a direct effect of the training on WM abilities in children with pSD. This effect translates into large and significant improvements in CogMed indices and in an more active WM task requiring to maintain and process information in memory during a fine motor task, abilities called for the Following Instruction test. Thus, children with pSD increased both memory span, that is the number of units maintained in memory, and updating, the ability to control and actively manipulate information held for a short time in memory. These results, in agreement with Diamond's recommendation of continually challenging WM (6), suggest that an intensive and automatically adjusted training may be proposed to children with pSD to improve both storage and rapid updating. No significant differences emerged between pre- and post- training assessment at the CogMed rating scale questionnaires. We can speculate that given these questionnaires were tailored for children with ADHD, they may not be sufficiently sensible to detect changing in executive function and attention difficulties associated to a neurological condition. Our findings showed a generalization of the CogMed effects to other neuropsychological processes not directly targeted by the training, some of which represent areas of weakness in children with pSD, in particular visuo-spatial and executive functions. Significant improvements, indeed, were found in visuo-spatial tasks requiring visuo-construction abilities and in executive functioning, in terms of increased speed in inhibition and in improvements in phonological WM. These results are in agreement with several studies documenting CogMed cascade effects on untrained skills in everyday functions and on the core deficit of a certain neurodevelopment disorder [for a systematic review (13, 81)].

Although these findings support, as highlighted by some authors (82), that the generalization effects of a WM training tend to mainly involve the components within the EF domain directly engaged in the training, they provide new and relevant insights for implementing cognitive rehabilitation strategies in children with pSD. In fact, WM is a transversal cognitive function, important for reasoning, comprehension and learning, which may influence cognition across-the-board and induce cascade improvements on several neuropsychological processes (83, 84). The finding of transfer effects to other impaired functions is particularly important in children with pSD where the neuropsychological impairment may have a multi-level organization (36). Indeed, a training on WM may have direct effects on the EF impairment found in more than 50% of the children with pSD (36), and, at the same time, it may indirectly reduce visuo-spatial deficits, extensively documented in this clinical condition (26, 34).

In order to further understand how working memory training affects performance in children with pSD, the study analyzed whether the training effects were correlated to the clinical characteristics of the sample. No correlations were found between chronological and gestational ages, intelligence level or gross motor functioning and on the trained WM outcomes (CogMed improvement index and Following the Instructions tests). These findings are in agreement with previous evidence describing gains in WM capacity after CogMed training in different clinical populations (56, 57), and across different ages (48, 55, 63, 78, 79, 85), and support that, within a specific CP form, improvements may be found regardless of clinical variability such as different levels of motor deficit, grades of prematurity and intelligence quotients, the latter, within the normal range.

Nevertheless, improvements in the untrained tasks were found to be variably related to clinical characteristics. The gross-motor functions severity was related to the improvement in visuo-motor tasks, visuo-spatial memory and visuo-perceptual abilities: as severity of gross motor impairment increased, children showed greater gains in visuo-motor and visual memory skills but smaller improvements in a visuo-perceptual task. On the basis of these findings, although exploratory and based on a small group, one could speculate that the different generalization effects of the WM training on the neuropsychological processes found impaired in children with pSD, is related to the degree of gross motor disabilities.

Some methodological limitations of the present study should be pointed out. The data are based on a small sample of children with pSD due to periventricular leukomalacia and with average verbal intelligence, thus its findings must be confirmed in larger school-age samples, all trained with the same CogMed version. Moreover, since we used a comprehensive neuropsychological battery (NEPSY-II) to test EFs, a more fine-grained analysis of specific processes and components accounting for performance may have been highlighted, if specific EF tasks had been implemented.

In spite of these limitations, the study underlines the importance for integrating cognitive trainings focused on EF in the rehabilitation schedules of children with pSD which are most frequently focused only on motor/psychomotor interventions.

In conclusion, this study suggests that a home-based working memory training in children with pSD has a beneficial effect on trained working memory tasks as well as a generalization effect on other visuo-spatial and executive function tasks, especially for those subcomponents requiring cognitive control and updating. This study extends Grunewaldt et al.'s results in premature children showing beneficial training effects also in premature children with cerebral palsy.

Although Randomized Control Trial longitudinal studies on larger sample, with statistically more rigorous and robust comparisons of primary and secondary outcomes are needed to confirm these findings, it is suggested that a home-based WM training is an effective intervention for children with pSD which as it contributes to reducing hospital stays, already prolonged for motor assessments and interventions, and preventing cognitive weaknesses negatively impacting educational achievement and social functions.

## Data Availability Statement

The raw data supporting the conclusions of this article will be made available by the authors, without undue reservation.

## Ethics Statement

The studies involving human participants were reviewed and approved by Ethical Committee of the Fondazione Stella Maris Institute. Written informed consent to participate in this study was provided by the participants' legal guardian/next of kin.

## Author Contributions

MD: substantial contribution to the conception and design, acquisition, analysis and interpretation of data, and drafting. CP: substantial contribution to the conception and design, critical revision for important intellectual content, analysis and interpretation of data, and drafting. PB: substantial contribution to the conception and design, critical revision for important intellectual content analysis and interpretation of data, and drafting. GS: critical revision for important intellectual content analysis, accountable for ensuring questions related to accuracy or integrity of any part of the work are appropriately investigated and resolved, and final approval. MD'O, SP, and ES: acquisition, analysis and interpretation of data. AC: substantial contribution to the conception and design and critical revision for important intellectual content. AG: critical revision for important intellectual content. GC: critical revision for important intellectual content, accountable for ensuring questions related to accuracy or integrity of any part of the work are appropriately investigated and resolved, and final approval. All authors contributed to the article and approved the submitted version.

## Conflict of Interest

The authors declare that the research was conducted in the absence of any commercial or financial relationships that could be construed as a potential conflict of interest.
